# Prevalence of prediabetes by the fasting plasma glucose and HbA1c screening criteria among the children and adolescents of Shenzhen, China

**DOI:** 10.3389/fendo.2024.1301921

**Published:** 2024-01-19

**Authors:** Chen Yang, Shaohua Li, Liangyan Wu, Zan Ding, Hua Zhou, Ying Pan, Chufu Yang, Jinjun Lin, Qiang Li, Yingbin You, Xu Zhong, Yuyuan Chen, Yu Zhao

**Affiliations:** ^1^ Department of Endocrinology, Baoan Central Hospital of Shenzhen, The 5th Affiliated Hospital of Shenzhen University Health Science Center, Shenzhen, Guangdong, China; ^2^ Department of Endocrinology and Metabolism, Zhuhai People's Hospital (Zhuhai Clinical Medical College of Jinan University), Zhuhai, Guangdong, China; ^3^ Wards of Cadres, Zhuhai People's Hospital (Zhuhai Clinical Medical College of Jinan University), Zhuhai, Guangdong, China; ^4^ Department of Endocrinology and Metabolism, The First Affiliated Hospital of Jinan University, Guangzhou, China; ^5^ Department of Science and Education, Baoan Central Hospital of Shenzhen, Shenzhen, Guangdong, China; ^6^ Department of Administrative, Baoan Central Hospital of Shenzhen, Shenzhen, Guangdong, China; ^7^ Department of Huangtian Outpatient, Baoan Central Hospital of Shenzhen, Shenzhen, Guangdong, China; ^8^ Department of Endocrinology, Shenzhen University General Hospital, Shenzhen, Guangdong, China

**Keywords:** adolescents, children, prediabetes, prevalence, Shenzhen

## Abstract

**Background:**

Prediabetes is associated with an increased risk of cardiovascular diseases and all-cause mortality. Rare research in China has evaluated the prevalence of prediabetes among children and adolescents using the HbA1c criterion or the combined FPG-or-HbA1c diagnostic criterion, and researchers paid no attention to the distributions of blood glucose in Shenzhen, especially for juveniles.

**Methods:**

We conducted a school-based cross-sectional study based on the first-year students from 17 primary, middle, and high schools. Prediabetes was defined as FPG of 5.6–6.9 mmol/L or HbA1c of 5.7%–6.4%. The crude and standardized prevalence of prediabetes with 95% confidence interval (95% CI) was estimated.

**Results:**

A total of 7519 participants, aged 6 to 17 years, were included. For all subjects, the crude prevalence (95% CI) of prediabetes was 1.49% (1.21–1.77), 8.72% (8.08–9.36), and 9.80% (9.13–10.47) by the FPG-only, HbA1c-only, and FPG-or-HbA1c criteria, respectively. Based on the 2010 Shenzhen census population, the standardized prevalence was 1.56% (males 1.85%, females 1.19%), 11.05% (males 11.47%, females 10.53%), and 12.19% (males 13.01%, females 11.15%) by the corresponding criteria. The proportion of prediabetes was higher for males than females, and the prevalence decreased with grade for males but increased for females. The association of BMI and prediabetes was U-shaped curve, indicating higher rates of prediabetes for underweight and obesity people.

**Conclusion:**

The blood glucose status of children and adolescents in Shenzhen is worrisome, and the early detection and management of prediabetes are imperative.

## Highlight

- Our results showed a high crude/standardized rate of prediabetes among the children and adolescents of Shenzhen, China.
- The proportion of prediabetes was higher for males than females, and decreased with grade for males but increased for females.
- The association of BMI and prediabetes was U-shaped curve, indicating higher rates of prediabetes for underweight and obesity people.

## 1 Introduction

Defined as an intermediate metabolic state between normal-glycaemia and diabetes, prediabetes [those with impaired fasting glucose (IFG) and/or impaired glucose tolerance (IGT) and/or increased level of hemoglobin A1c (HbA1c)] is associated with an increased risk of CVD and all-cause mortality (1–4). Some studies found that vascular complications, nephropathy, retinopathy and neuropathies are more common in people with prediabetes than individuals at normal blood glucose levels (5–8).

The number of children and adolescents with type 2 diabetes has increased two to three times in the last 30 years (9). A national data of United States reported that the prevalence of prediabetes among adolescents in the US has more than tripled from 1999 to 2020 (10). In China, the estimated overall prevalence of diabetes and prediabetes among Chinese adults (18 years of age or older) in 2013 were 10.9% and 35.7% (11). In Chinese children and adolescents, the 2002 national data showed that the prevalence of diabetes was 0.19% (12), and the Beijing data displayed that the total age-adjusted prevalence of diabetes and IFG were 0.57% and 1.35% (13). Many studies on prediabetes were carried out in adults but few in children and adolescents. With the increasing of incidences of diabetes and prediabetes among children and adolescents, identifying children at risk and taking intervening measures can have an effective role in preventing CVD and reducing long-term mortality and morbidity (11–14).

There is a huge difference in eating habits and diet between China and other countries, and Chinese is mainly based on carbohydrates (15). Besides, because of the one-child policy and the rapid economic development economy of China in recent years, lifestyles and physical activities of Chinese children and adolescents have changed a lot than before (16). Thus, data from other countries could not reflect the situation of blood glucose level in China. Furthermore, no relevant survey in south China has updated the information about elevated blood sugar among children and adolescents in the last decade, and the prevalence of prediabetes has not yet been investigated among children and adolescents in the Pearl River Delta of Guangdong province (e.g., Shenzhen, a rapidly rising new city with fairly developed economy).

Some methodological studies indicated that the HbA1c criterion was associated with a higher diagnosis rate of prediabetes compared to the FPG or oral glucose tolerance test (OGTT) in non-diabetic children and adults (17, 18). Combined application of HbA1c and FPG test in screening for prediabetes reduces the risk of systematic bias inherent and provides the benefits of individual test in using only one test (17, 18). However, no research in China evaluated the prevalence of prediabetes among children and adolescents using the HbA1c criterion or the combined FPG-or-HbA1c diagnostic criterion. Reliable surveillance data are crucial for the identification, surveillance, and characterization of high-risk populations, which are necessary for the planning of interventions and assessing the effectiveness of diabetes prevention strategies (19, 20). Thus, we conducted a cross-sectional observational survey to examine the newly crude and standardized prevalence of prediabetes for the total group as well as the sub-groups by grade and sex among the children and adolescents of Shenzhen, China in 2017, based on the FPG-only, HbA1c-only, and the combined FPG-or-HbA1c diagnostic criteria.

## 2 Materials and methods

### 2.1 Study subjects and design

Located in the Pearl River Delta region in southern China, Shenzhen is the first Special Economic Zone of China and the largest manufacturing base in the world, with a total gross domestic product (GDP) of 1.75 trillion CNY (i.e. 0.27 trillion USD) and 157,985 CNY (i.e. 24,640 USD) per capita in 2015. The total area of Shenzhen is 1997.3 km^2^, and the population is around 11.37 million with about 70% being migrants (21). Among 10 district-level jurisdictions of Shenzhen, Baoan district is accounted for the first largest area, with a total population of 2.86 million at the end of 2016, accounting for a quarter of the total population of Shenzhen (21). Exclusion criteria were patients with severe diseases or having cognitive, language, or mental disorders who could not participate in the subject. No students had been excluded.

### 2.2 Measures and data collection

Based on the compulsory admission physical examination organized by the Baoan District Government of Shenzhen, we conducted a school-based cross-sectional study recruited all the freshmen from the grade 1 of the primary school, middle school, and high school (i.e., the grade 1, 7, and 10, respectively) in the Baoan District of Shenzhen between February and June 2017. The stratified cluster sampling method was adopted to randomly select representative schools from different regions of the district. In following, 17 elementary schools for girls and boys were selected. Subsequently, with respect to the population of the respective school and the class, a number of students were chosen from each class. Based on the above procedure, 7800 students from elementary, middle and high school were selected. This research program was approved by the Ethical Review Committee of Baoan Central Hospital of Shenzhen. Written informed consent was obtained from all of the students and their parents.

Only certified staff were allowed to take part in this study. Height and weight were measured by trained physicians to the nearest 0.1cm and 0.1kg, without shoes and in light clothing. Individual characteristic data including age, sex, and grade were double entered in a database manually. After an overnight fast of 10h, blood samples were drawn from an antecubital vein in each subject into vacutainer tubes. The tubes were delivered to laboratory of hospital in an insulated box with ice by a specially-assigned physician and centrifuged within an hour. FPG concentration was measured using the hexokinase method with the automatic analyzer (Model 7170s; Hitachi, Tokyo, Japan) by the Glucose Hexokinase FS, which was manufactured by Diasys Diagnostic Systems (Shanghai) CO, LTD. HbA1c was measured utilizing the ion high performance liquid chromatography (HPLC) with Bio-Rad VARIANT II (Made in USA), and the method is certified by the NGSP and standardized to the Diabetes Control and Complications Trial (DCCT) reference assay. All of the detection equipment in our laboratory have passed the National Center for Clinical laboratories external quality assessment.

### 2.3 Statistical analysis

Diabetes was defined as FPG of ≥ 7.0 mmol/L (126 mg/dL) or HbA1c of ≥ 6.5% (48 mmol/mol). Prediabetes was defined as FPG of 5.6 mmol/L (100 mg/dL) to 6.9 mmol/L (125 mg/dL) (IFG) or HbA1c of 5.7% to 6.4% (38.8 to 46.5 mmol/mol). Based on the international norms from the WHO with age (to the nearest one month) and gender-specific body mass index (BMI), BMI cutoffs were the following: overweight, BMI >1 SD; obesity, BMI >2SD; thinness, BMI < -2SD; and severe thinness, BMI < -3SD (SD was standard deviation of the BMI based z-scores) (22).

Continuous variables were expressed as mean ± SD. The prevalence of prediabetes with 95% confidence interval (95% CI) was estimated. Significance for inter-group difference in the prevalence of prediabetes was assessed by the Pearson’s chi-square test. Scatter charts and Spearman correlation analyses were used to evaluate the association of FPG with HbA1c values. A chi-square test for the trend of the crude grade-prevalence of prediabetes by sex was also performed. Age- and/or sex-standardized prevalence rates of prediabetes were estimated according to the distribution of the population in the 2010 Shenzhen population census as well as the Chinese population census by the direct standardization method. A multiple logistic regression analysis was performed, expressed as odds ratios and 95% CIs for risk of prediabetes. The dependent variable was the diagnoses of prediabetes (based on the FPG-or-HbA1c diagnostic criterion), and the independent variables were gender, grade, and classification of BMI based Z-scores. Statistical analyses and graphics involved use of SPSS for Windows 13.0 (SPSS Inc., Chicago, IL, USA) and SigmaPlot software version 10.0. Two-tailed tests of significance were reported, with *P* < 0.05 considered statistically significant.

## 3 Results

### 3.1 Sample characteristics

**Table 1** presents the summary statistics for FPG and HbA1c among the children and adolescents of Shenzhen by grade, sex, and the classification of BMI. Data were available for 7519 participants, aged 6 to 17 years, of which 4196 (55.8%) were males and 3323 (44.2%) were females. A total of 4785, 850, and 1884 participants were separately involved in the grade 1, 7, and 10, respectively. Overall, the mean (SD) and median (interquartile range [IQR]) of FPG were 5.28 (0.30) and 5.3 (5.1–5.5) mmol/L; the mean (SD) and median (IQR) of HbA1c level were 4.71% (0.40) and 4.7% (4.5–5.0), with a maximum value of 9.1%.

**Table 1:** Summary statistics for FPG and HbA1c in the children and adolescents of Shenzhen, China in 2017.

	No. of participants	FPG (mmol/L)		HbA1c (%)	
		Mean ± SD	Min–Max	Mean ± SD	Min–Max
Overall					
Total	7519	5.28 ± 0.30	3.00–7.30	4.71 ± 0.40	2.10–9.10
Males	4196	5.28 ± 0.32	3.00–7.30	4.76 ± 0.41	2.10–9.10
Females	3323	5.27 ± 0.28	3.90–6.40	4.65 ± 0.39	2.70–6.50
Grade 1 (6–10 years)					
Total	4785	5.28 ± 0.30	3.00–6.60	4.73 ± 0.39	2.10–6.70
Males	2680	5.29 ± 0.32	3.00–6.60	4.80 ± 0.38	2.10–6.70
Females	2105	5.27 ± 0.28	3.90–6.20	4.62 ± 0.38	2.70–6.30
Grade 7 (9–16 years)					
Total	850	5.29 ± 0.29	4.10–7.30	4.78 ± 0.38	3.50–9.10
Males	492	5.29 ± 0.31	4.10–7.30	4.78 ± 0.40	3.50–9.10
Females	358	5.28 ± 0.27	4.50–6.00	4.77 ± 0.35	3.50–5.70
Grade 10 (12–17 years)					
Total	1884	5.26 ± 0.31	3.80–6.40	4.65 ± 0.43	2.70–7.30
Males	1024	5.25 ± 0.32	3.80–6.40	4.64 ± 0.45	2.70–7.30
Females	860	5.27 ± 0.29	4.10–6.40	4.67 ± 0.41	3.40–6.50
Classification of BMI					
Missing	127	5.26 ± 0.32	4.20–6.10	4.67 ± 0.39	3.80–5.80
Thinness or severe thinness	236	5.33 ± 0.31	4.20–6.20	4.60 ± 0.41	3.10–5.50
Normal	5823	5.28 ± 0.30	3.00–6.60	4.70 ± 0.40	2.10–6.70
Overweight	896	5.26 ± 0.30	3.90–6.00	4.77 ± 0.39	2.80–7.30
Obesity	437	5.29 ± 0.33	4.10–7.30	4.80 ± 0.42	3.60–9.10

FPG, fasting plasma glucose; HbA1c, hemoglobin A1c; SD, standard deviation; Min–Max, Minimum–Maximum.

### 3.2 Correlation analysis

**Figure 1:**
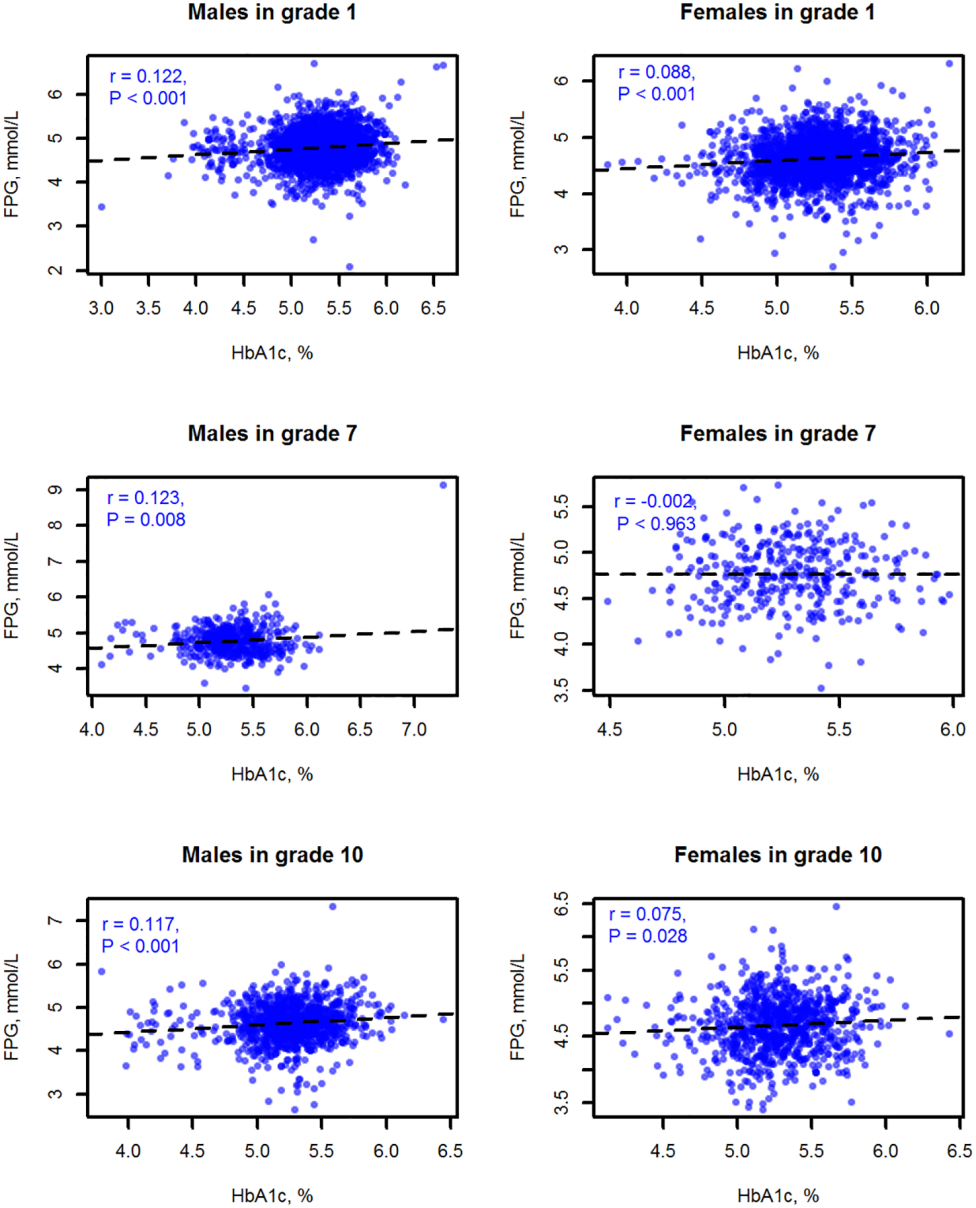
Scatter charts and Spearman correlation analyses of FPG with HbA1c values among the children and adolescents of Shenzhen by sex and grade.

The results of the correlation analysis showed that FPG and HbA1c correlated weakly. The Spearman correlation coefficient was 0.109 for all subjects, 0.122 for total males, and 0.088 for total females (all *P*<0.001). **Figure 1** displays the scatter charts of FPG with HbA1c values by sex and grade. In the stratification analyses, all correlations were positive and significant except the correlation of FPG with HbA1c among females in the grade 7 (r = -0.002, *P* = 0.963).

### 3.3 Crude prevalence of diabetes and prediabetes

A total of 4 students (all of them are males) were classified as having diabetes by the FPG-or-HbA1c criterion with a rate of 0.53‰ (4/7519), 2 from the grade 1, 1 from the grade 7, and 1 from the grade 10.

For all subjects, the crude prevalence (95% CI) of prediabetes was 1.49% (1.21–1.77), 8.72% (8.08–9.36), and 9.80% (9.13–10.47) by the FPG-only, HbA1c-only, and FPG-or-HbA1c criteria, respectively (**Table 2**). The prevalence of prediabetes was higher for males than females by the FPG-only (1.92% *vs* 0.94%; *P*=0.001) and FPG-or-HbA1c criteria (10.51% *vs* 8.10%; *P* < 0.001) but no significant difference by the HbA1c-only criterion (9.09% *vs* 8.25%; *P* = 0.198). There were no statistically differences among the prevalence by grade (all *P*>0.05). The association between BMI and the prevalence of prediabetes was shown to be non-linear and following a U-shaped curve (**Supplementary Figure S1**). By the HbA1c-only criterion, the BMI-based prevalence of prediabetes was slightly higher among the thinness or severe thinness (13.19%) and obesity (9.40%) than the normal group (8.68%) (**Table 2**).

**Table 2:** Crude prevalence of prediabetes among the children and adolescents of Shenzhen, China in 2017.

	FPG-only criterion	HbA1c-only criterion	FPG-or-HbA1c criterion
Overall	1.49 (1.21–1.77)	8.72 (8.08–9.36)	9.80 (9.13–10.47)
Sex			
Males	1.92 (1.50–2.35)	9.09 (8.22–9.96)	10.51 (9.58–11.44)
Females	0.94 (0.60–1.27)	8.25 (7.31–9.18)	8.10 (7.17–9.02)
*P* value for difference	0.001	0.198	< 0.001
Grade			
Grade 1	1.57 (1.21–1.94)	8.89 (8.08–9.7)	9.99 (9.14–10.84)
Grade 7	1.23 (0.47–1.99)	9.29 (7.34–11.25)	10.35 (8.30–12.4)
Grade 10	1.38 (0.86–1.91)	8.03 (6.80–9.26)	9.08 (7.78–10.37)
*P* value for difference	0.692	0.440	0.448
Classification of BMI			
Thinness or severe thinness	0 (0–0)	13.19 (8.86–17.52)	13.14 (8.83–17.45)
Normal	1.49 (1.17–1.8)	8.68 (7.95–9.40)	9.72 (8.96–10.48)
Overweight	1.99 (1.05–2.92)	7.73 (5.98–9.48)	9.38 (7.47–11.28)
Obesity	1.20 (0.16–2.25)	9.40 (6.66–12.14)	10.3 (7.45–13.15)
*P* value for difference	0.161	0.064	0.347

FPG, fasting plasma glucose; HbA1c, hemoglobin A1c. The prevalence of prediabetes is expressed as % (95% confidence interval).

**Figure 2:**
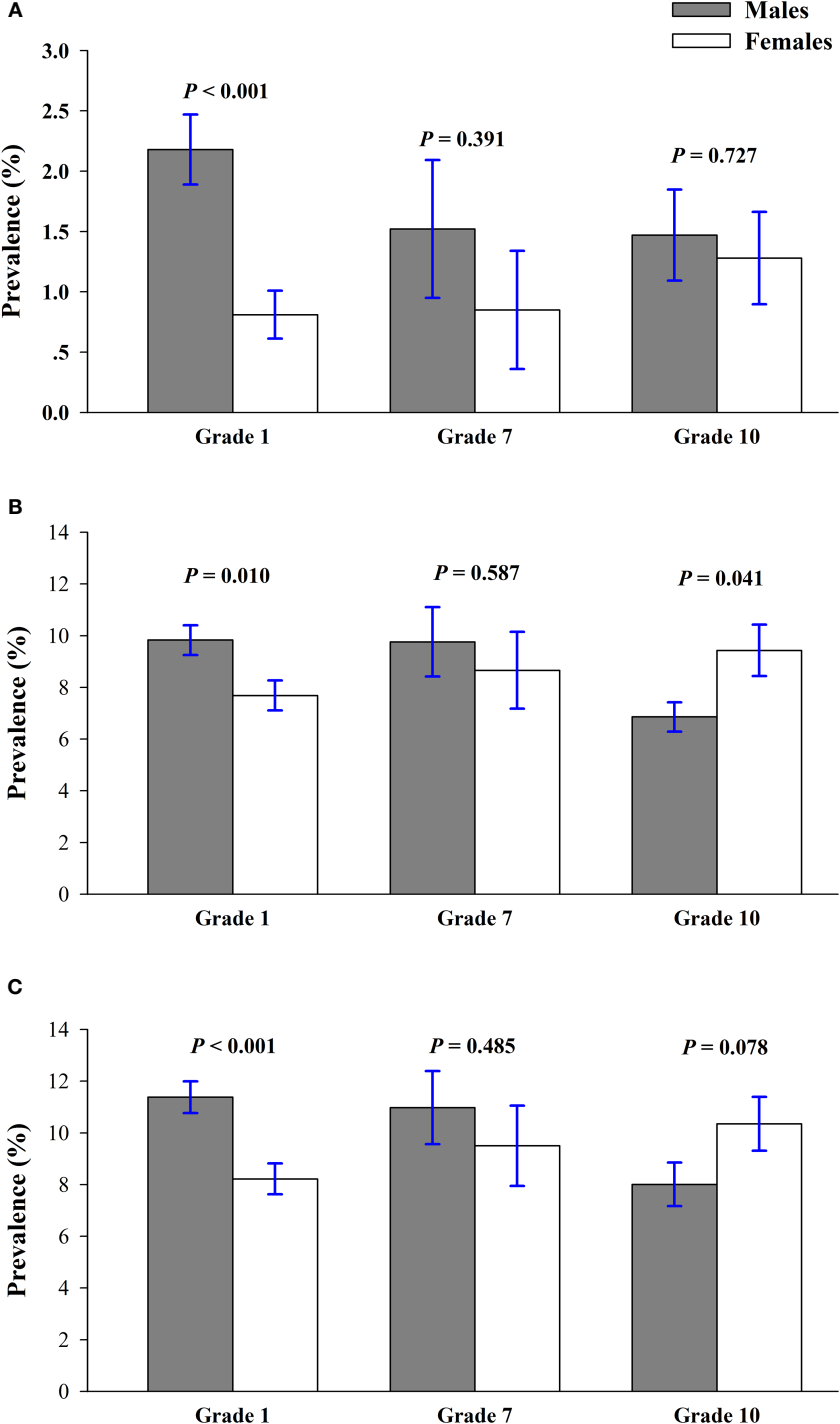
Crude sex-prevalence of prediabetes by grade among the children and adolescents of Shenzhen, China in 2017, according to FPG-only **(A)**, HbA1c-only **(B)**, and the combined FPG-or-HbA1c **(C)** diagnostic criteria.

**Figure 3:**
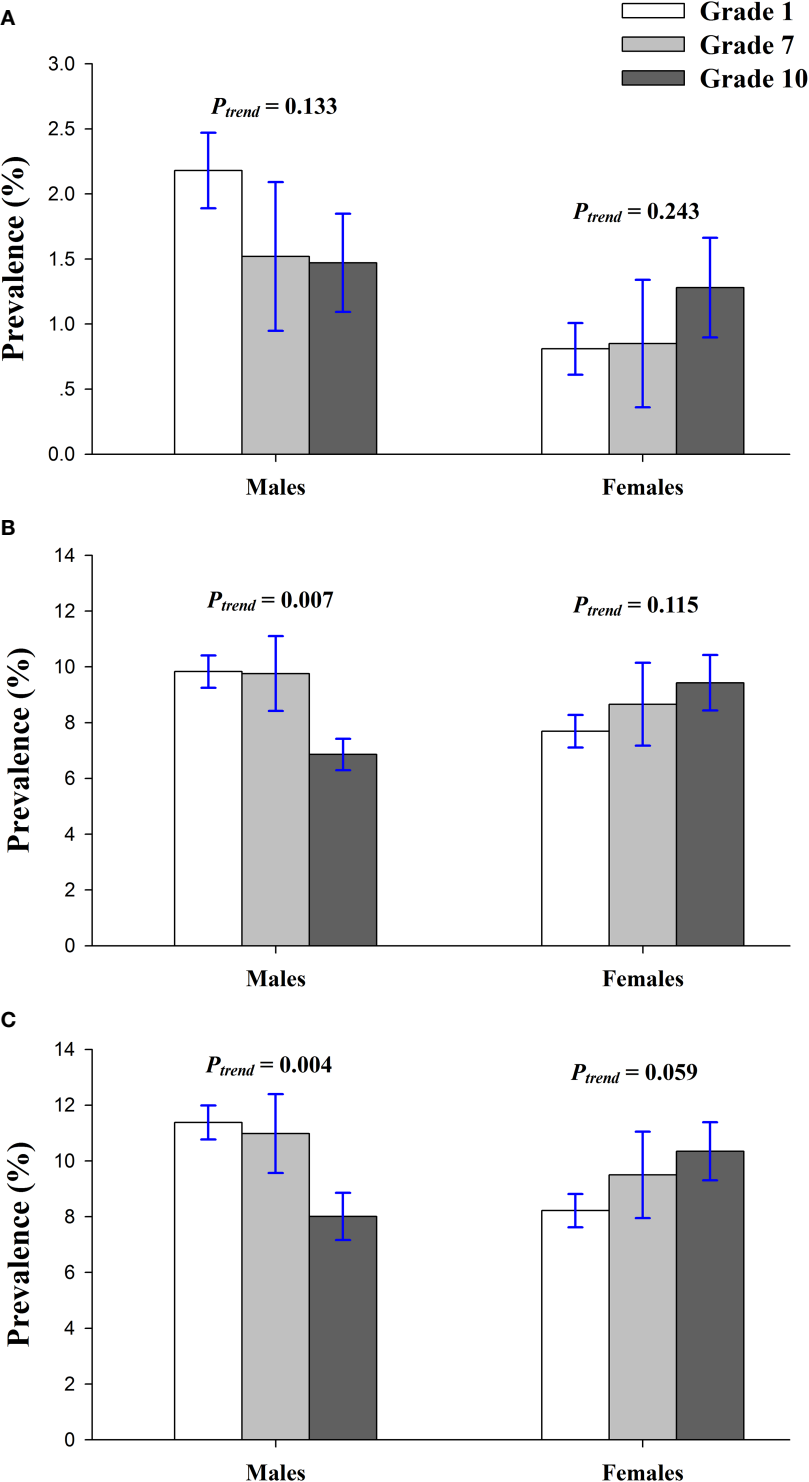
Crude grade-prevalence of prediabetes by sex among the children and adolescents of Shenzhen, China in 2017, according to FPG-only **(A)**, HbA1c-only **(B)**, and the combined FPG-or-HbA1c **(C)** diagnostic criteria.

**Figure 2** shows the crude sex-prevalence of prediabetes by grade. A greater proportion of boys than girls had an elevated FPG, though this difference was statically significant only in the grade 1, the youngest children, where 2.18% of boys but only 0.81% of girls had FPG of 5.6 mmol/L or greater (*P* < 0.01). According to the HbA1c-only criteria, the prevalence of prediabetes was higher among males compared with females in the grade 1 (9.83% *vs* 7.69%; *P*=0.010) but higher for females than males in the grade 10 (6.86% *vs* 9.43%; *P* = 0.041). Depending on the FPG-or-HbA1c criterion, females had a higher prevalence than males in the grade 1, but the trend was adverse in the grade 10 (8.01% *vs* 10.35%; *P* = 0.078). **Figure 3** suggested that prevalence of prediabetes dwindled with increasing grade/age for males by the FPG-only, HbA1c-only, and FPG-or-HbA1c criteria (*P* = 0.130, 0.007, and 0.004, respectively), but the prevalence increased with grade for females by the 3 diagnose criteria, though this trend has no statistically significant difference (all *P*trend > 0.05).

### 3.4 Standardized prevalence of prediabetes

**Figure 4:**
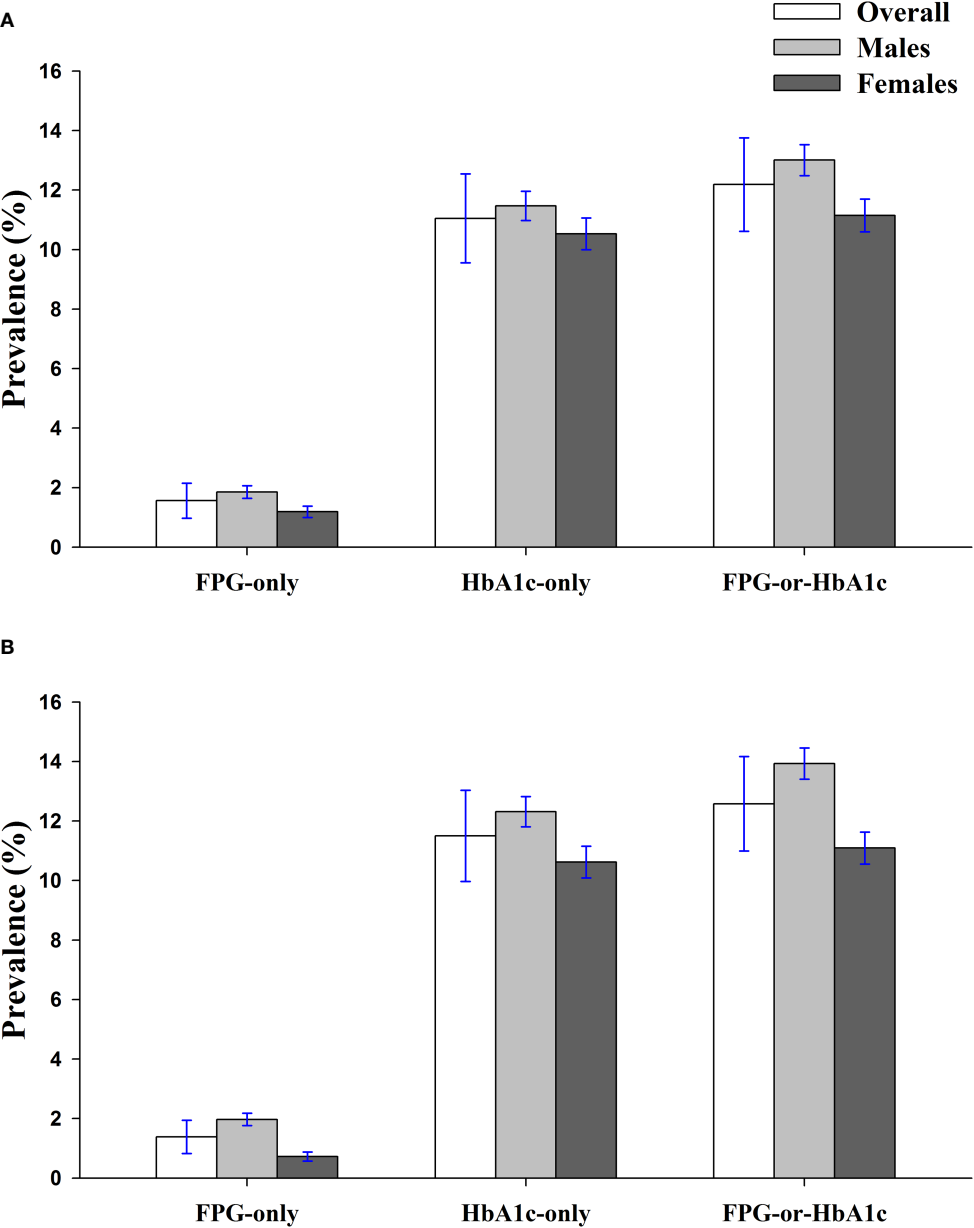
Standardized prevalence of prediabetes among the children and adolescents of Shenzhen, China in 2017 by the FPG-only, HbA1c-only, and FPG-or-HbA1c criteria, based on the 2010 Shenzhen Census Population **(A)** and 2010 Chinese National Census Population **(B)**.

Based on the 2010 Shenzhen census population, the age- and sex-standardized prevalence (95% CI) of prediabetes were 1.56% (1.28–1.84), 11.05% (10.34–11.76), and 12.19% (11.45–12.93) by the FPG-only, HbA1c-only, and FPG-or-HbA1c criteria, respectively (**Figure 4**). The age-standardized prevalence of prediabetes was more prevalent in males than females by the corresponding criteria (*P* = 0.023, 0.201, and 0.015, respectively). Based on the 2010 Chinese census population, the age- and sex-standardized prevalence of prediabetes was 1.38% (1.12–1.64), 11.50% (10.78–12.22), and 12.58% (11.83–13.33) by the FPG-only, HbA1c-only, and FPG-or-HbA1c criteria, respectively. The age-standardized rates of prediabetes among males and females were 1.97% and 0.72%, 12.31% and 10.62%, 13.93% and 11.09% by FPG-only, HbA1c-only, and FPG-or-HbA1c, respectively (**Figure 4**).

### 3.5 Multivariable logistic regression analysis

Our regression analysis in **Table 3** revealed that the proportion of prediabetes by the FPG-or-HbA1c criterion was higher for males than females with an odds ratio (95% CI) of 1.21 (1.03–1.41), adjusted for grade and classification of BMI. However, no differences in prevalence of prediabetes among the sub-groups separated by the grade or BMI categories were shown (all *P*>0.05), consistent with the findings in **Table 2** based on the univariate analyses.

**Table 3:** Multivariable logistic regression analysis with prediabetes diagnosed by the FPG-or-HbA1c criterion as outcome variable.

Variable	Wald test	Odds ratio (95% CI)	*P*
Males	5.42	1.21 (1.03–1.41)	0.020^*^
Grade	1.89	–	0.389
Grade 1	0.04	1.00	–
Grade 7	1.67	1.02 (0.80–1.30)	0.848
Grade 10	3.11	0.89 (0.74–1.07)	0.196
Classification of BMI	0.14	–	0.375
Thinness or severe thinness	0.02	1.40 (0.95–2.06)	0.092
Normal	2.84	1.00	–
Overweight	5.42	0.96 (0.75–1.21)	0.710
Obesity	1.89	1.02 (0.74–1.41)	0.903
Constant	1100.81	0.101	<0.001^*^

^*^P < 0.05.

## 4 Discussion

### 4.1 A general run of this study

Regarded as the “most vigorous and the youngest city”, Shenzhen has an advanced economy and offers a large number of employment opportunities, attracting many young immigrants aged 18–40 years. Because of higher levels of education and the pursuit of higher quality of life, many young people choose late marriage and childbearing. Children and adolescents have always been a group of the great concern to society, and the government of Shenzhen attaches a great importance to their healthy growth, including strengthening education and physical health. However, researchers paid no attention to the distributions of blood glucose in Shenzhen, especially for juveniles.

Covering 7519 children and adolescents of Shenzhen during 2017, our school-based study firstly estimated the crude and standardized prevalence of prediabetes based on the FPG-only, HbA1c-only, and FPG-or-HbA1c diagnostic criteria. The results showed a high crude/standardized rate of prediabetes. The proportion of prediabetes was higher for males than females, and the prevalence decreased with grade for males but increased for females.

### 4.2 An overview on the prevalence of prediabetes

Considered prediabetes as having FPG in 5.6–7.0 mmol/l (i.e. IFG) or HbA1c in 5.7–6.4%, we found that the Shenzhen-standardized prevalence of prediabetes was 12.19% (13.01% for males and 11.15% for females; by FPG-or-HbA1c criterion). This rate exceeded that of several Chinese regions such as Beijing (1.35%; aged 6–18 years (13), Hebei (3.5% for total, 3.9% for males, and 3.1% for females; aged 13–18 years) (23), and Xinjiang (0.7%; aged 0–17 years) by the FPG-only criterion (24). However, the rate of Shenzhen was significantly lower than that of American adolescents (40.5%, by FPG-only and/or 2-h glucose; aged 12–14 years) (25), Emirati (21.9%, by combined FPG and HbA1c, aged 11–17 years) (26), Mexico (18.3%, by FPG-only and/or 2-h glucose; aged 6–18 years) (27), the USA (16.1% for total, 22.4% for males, and 9.5% for females; by FPG-only or 2-h glucose; aged 12–19 years) (28). The differing prevalence rates may result from variations in the sampling method, participant age, ethnicity, region, sampling size, diagnostic criteria, and research period (27, 29).

### 4.3 FPG and HbA1c diagnostic criteria

Our study presented that HbA1c values were weakly correlated with FPG values, especially for females. Several other surveys on the diadynamic criteria of diabetes and prediabetes among minors or adults also revealed similar findings (19, 30–32). For instance, a retrospective study among German children and adolescents showed a poor correlation of FPG with HbA1c (r = 0.18) (32), and a Korean study also disclosed that the kappa coefficient of agreement between FPG and HbA1c was 0.396 (95% CI, 0.356–0.459) in obese children and adolescents (31).

Compared with FPG and OGTT diagnostic criteria, HbA1c criterion is greater convenience (fasting not required), greater preanalytical stability, and less day-to-day perturbations during stress and illness. However, several disadvantages of HbA1c testing are the lower sensitivity at the designated cut-point, higher cost, limited availability in certain regions of developing countries, and the imperfect correlation between HbA1c and average glucose in certain individuals. In 2010, American Diabetes Association published revised and modified diagnostic guidelines recommending that HbA1c tests could be used for diagnosing diabetes (≥6.5% or ≥48 mmol/mol) and prediabetes (5.7–6.4% or 38–47 mmol/mol) in both adults and children (33). However, whether the HbA1c method was applicable to children and adolescents for screening of diabetes and prediabetes was still controversial up to now. Several studies indicate that using adult cut-off points of HbA1c to predict prediabetes or diabetes significantly underestimates the prevalence of these conditions in the pediatric and adolescent population, and consequently a lower HbA1c cut-off point should be proposed for children (26, 34–36). However, the Emirati research indicated a significant higher proportion of children with prediabetes by using the HbA1c than OGTT criterion (21.9% *vs* 5.4%), and our study also showed a higher prevalence by HbA1c than FPG criterion (8.72% *vs* 1.49%). When using HbA1c to screen diabetes or prediabetes, it is important to recognize that HbA1c is an indirect measure of average blood glucose levels and to take other factors into consideration that may impact hemoglobin glycation independently of glycaemia, including age, race/ethnicity, and anemia/hemoglobinopathies (37). Thus, it remains unclear if HbA1c and its cut-off points for adults could be used to diagnose diabetes and prediabetes in children and adolescents, even in Chinese pediatric and youth. Our research provided a reference and proposed more studies to fully assess the impact of using the HbA1c criterion and to formulate the age- and race/ethnicity-specific cut-off points for pediatric and adolescent population.

### 4.4 Prevalence of prediabetes by gender

Our study revealed that males among children and adolescents of Shenzhen exhibited a higher prevalence of IFG compared with that of females, supported by the reported results of a series of international studies (28, 29, 38, 39). However, several studies have found no gender differences regarding IFG prevalence in children and adolescents (23, 40). The proportion of elevated HbA1c showed an obvious sex difference, which was not consistent with a majority of surveys (41–43). The current study also demonstrated that the prevalence of prediabetes decreased with grade/age for males but increased for females, but these trends can scarcely be seen in no matter adults or children in other studies (41–43). The underlying mechanisms for the variation of prevalence by gender and grade/age are not quite clear. It might be linked to the child’s growth and multiple hormonal changes effective on different metabolic patterns (43, 44).

### 4.5 Prevalence of prediabetes by BMI

A recent large prospective cohort of 12.8 million adults demonstrated a U-shaped association between BMI and total-mortality in diabetes, regardless of diabetes status in both sexes and all age-groups (45). In young-onset diabetes, normal weight and underweight contributed more to reduced longevity compared with overweight and obesity (45). Numerous studies also reported various types (U-curve, L-curve, or inverse linear) of BMI-mortality associations in diabetes (45–47). Our observation on the association between BMI and the prevalence of prediabetes was shown to be non-linear and following a similar U-shaped curve, which indicated higher rates of prediabetes for underweight and obesity people. Because the hyperglycemia of children and adolescents is a major health issue requiring immediate action, further research to better understand the underlying mechanisms are needed.

### 4.6 Prevention of progression from prediabetes to diabetes

The average age of onset for diabetes has gradually declined. A cross-sectional survey of a nationally representative sample of 98,658 Chinese adults in 2010 revealed a high prevalence of prediabetes in the younger age groups, which may translate into a greater epidemic of diabetes in the near future (42). It has been suggested that the progression is faster for children and adolescents than adults, with more rapid deterioration of β-cell function (48–50). Findings from our study suggested a great number of children and adolescents of Shenzhen will join the diabetes crowds over 2–10 years without an effective national intervention (e.g. extensive epidemiological surveys, professional health education, and intervention guides) (51–53). A diabetes epidemic would further burden an already overloaded health care system in China. The health care costs for diabetes would likely become a huge financial burden on patients and their families and society as whole (42, 54).

A prospective, longitudinal study conducted by Yale University School of Medicine showed that up to 50% of severely obese adolescents with prediabetes subsequently reverted to normal glucose tolerance, whereas 24% progressed from prediabetes to diabetes (48). Several randomized and controlled trials involving adults with IGT, some of whom also had IFG, have also provided evidence that lifestyle changes (e.g. increased physical activity and dietary changes) or metformin can significantly reduce the incidence of diabetes (38, 55–57). Thus, health promotion plans should be actively established for this population group to facilitate early prevention or delay the onset of diabetes. In a supportive environment, the provision of nutrition education and appropriate food selection concepts should be enhanced for adolescents, and families should teach and monitor adolescents’ dietary behavior. Through lifestyle modifications, insulin sensitivity can be significantly improved to effectively prevent or delay the development of diabetes and relevant complications (58, 59).

## 5 Conclusions

The blood glucose status of children and adolescents in Shenzhen is worrisome. Males had a higher prevalence of prediabetes than females, and the prevalence decreased with grade for males but increased for females. The early detection and management of diabetes and prediabetes are imperative. These findings may help in better targeting the vulnerable students, developing more effective intervention strategies, and leading more researchers’ attention in this area.

## Data availability statement

The raw data supporting the conclusions of this article will be made available by the authors, without undue reservation.

## Ethics statement

The studies involving humans were approved by the Ethical Review Committee of Baoan Central Hospital of Shenzhen. The studies were conducted in accordance with the local legislation and institutional requirements. Written informed consent for participation in this study was provided by the participants’ legal guardians/next of kin.

## Author contributions

CY: Funding acquisition, Supervision, Writing – original draft. SL: Data curation, Investigation, Validation, Writing – original draft. LW: Formal analysis, Investigation, Writing – original draft. ZD: Writing – original draft, Writing – review & editing. HZ: Supervision, Writing – review & editing. YP: Investigation, Writing – review & editing. CY: Investigation, Writing – review & editing. JL: Investigation, Writing – review & editing. QL: Investigation, Writing – review & editing. YY: Investigation, Writing – review & editing. XZ: Investigation, Writing – review & editing. YC: Investigation, Writing – original draft. YZ: Supervision, Validation, Writing – review & editing.
