# Supplementary material for: Prevalence of prediabetes by the fasting plasma glucose and HbA1c screening criteria among the children and adolescents of Shenzhen, China
Source: Front Endocrinol (Lausanne). 2024 Jan 19;15:1301921. doi: 10.3389/fendo.2024.1301921 (PMC10836591; doi:10.3389/fendo.2024.1301921)
Supplement: Supplementary file 1 [file DataSheet_1.doc]

**Table S1. Main characteristics of cross-sectional epidemiological studies about the prevalence of prediabetes among children and adolescents.**

| **No.** | **Study** | **Target population** | **Age groups, years** | **Sample size** | **Diagnostic criterion** | **Prevalence of prediabetes** |
| --- | --- | --- | --- | --- | --- | --- |
| 1 | Present study, Shenzhen, China | All students | 6-17 | 7519 | FPG | 1.56% for total, 1.85% for males, and 1.19% for females. |
|  | Present study, Shenzhen, China | All students | 6-17 | 7519 | HbA1c | 11.05% for total, 11.47% for males, and 10.53% for females. |
|  | Present study, Shenzhen, China | All students | 6-17 | 7519 | FPG-or-HbA1c | 12.19% for total, 13.01% for males, and 11.15% for females. |
| 2 | Lu et al. (2007), Beijing, China | All students | 6–18 | 19593 | FPG | 1.35% for total |
| 3 | Baranowski et al. (2006), USA | Overweight/obese students | 12–14 | 1740 | FPG | 40.5% for total |
|  | Baranowski et al. (2006), USA | Overweight/obese students | 12–14 | 1740 | 2-h glucose | 2.0% for total |
| 4 | Al Amiri et al. (2015), Emirati | Overweight/obese | 11–17 | 1034 | 2-h glucose | 0.87% for total |
|  | Al Amiri et al. (2015), Emirati | Overweight/obese | 11–17 | 1034 | HbA1c | 21.9% for total |
| 5 | Guerrero-Romero et al. (2009), Mexican | All students | 6–18 | 1534 | FPG | 18.3% for total |
|  | Guerrero-Romero et al. (2009), Mexican | All students | 6–18 | 1534 | 2-h glucose | 5.2% for total |
| 6 | Cao et al. (2007), Hebei, China | All students | 13–18 | 3937 | FPG | 3.5% for total, 3.9% for males, and 3.1% for females |
| 7 | Li et al. (2009), USA | All students | 12–19 | 2288 | FPG | 13.1% for total |
|  | Li et al. (2009), USA | All students | 12–19 | 2288 | 2-h glucose | 3.4,% for total |
|  | Li et al. (2009), USA | All students | 12–19 | 2288 | FPG and/or 2-h glucose | 16.1% for total, 22.4% for males, and 9.5% for females |
| 8 | Zhang et al. (2013), Xinjiang, China | All students | 0–17 | 3644 | FPG | 0.7% for total, 0.7% for males, and 0.6% for females |
| 9 | Zhu et al. (2013), Tianjin, China | Overweight/obese | 7–18 | 727 | FPG and/or 2-h glucose | 3.3% for total |





**Figure S1.** Crude prevalence of prediabetes by classification of BMI based Z-scores among the children and adolescents of Shenzhen, China in 2017, according to the combined FPG-or-HbA1c diagnostic criterion.
